# The hidden financial burden of healthcare: a systematic literature review of informal payments in Sub-Saharan Africa

**DOI:** 10.12688/wellcomeopenres.17228.1

**Published:** 2021-11-08

**Authors:** Evelyn Kabia, Catherine Goodman, Dina Balabanova, Kui Muraya, Sassy Molyneux, Edwine Barasa

**Affiliations:** 1 Health Economics Research Unit, KEMRI Wellcome Trust Research Programme, Nairobi, Kenya; 2 Department of Global Health and Development, London School of Hygiene and Tropical Medicine, London, UK; 3 Health Systems & Research Ethics Department, KEMRI Wellcome Trust Research Programme, Nairobi, Kenya; 4 Health Systems & Research Ethics Department, KEMRI Wellcome Trust Research Programme, Kilifi, Kenya; 5 Center for Tropical Medicine and Global Health,Nuffield Department of Medicine, University of Oxford, Oxford, UK

**Keywords:** Informal payments, health, Sub-Saharan Africa, review

## Abstract

**Background: **Informal payments limit equitable access to healthcare. Despite being a common phenomenon, there is a need for an in-depth analysis of informal charging practices in the Sub-Saharan Africa (SSA) context. We conducted a systematic literature review to synthesize existing evidence on the prevalence, characteristics, associated factors, and impact of informal payments in SSA.

**Methods: **We searched for literature on PubMed, African Index Medicus, Directory of Open Access Journals, and Google Scholar databases and relevant organizational websites. We included empirical studies on informal payments conducted in SSA regardless of the study design and year of publication and excluded reviews, editorials, and conference presentations. Framework analysis was conducted, and the review findings were synthesized.

**Results: **A total of 1700 articles were retrieved, of which 23 were included in the review. Several studies ranging from large-scale nationally representative surveys to in-depth qualitative studies have shown that informal payments are prevalent in SSA regardless of the health service, facility level, and sector. Informal payments were initiated mostly by health workers compared to patients and they were largely made in cash rather than in kind. Patients made informal payments to access services, skip queues, receive higher quality of care, and express gratitude. The poor and people who were unaware of service charges, were more likely to pay informally. Supply-side factors associated with informal payments included low and irregular health worker salaries, weak accountability mechanisms, and perceptions of widespread corruption in the public sector. Informal payments limited access especially among the poor and the inability to pay was associated with delayed or forgone care and provision of lower-quality care.

**Conclusions:** Addressing informal payments in SSA requires a multifaceted approach. Potential strategies include enhancing patient awareness of service fees, revisiting health worker incentives, strengthening accountability mechanisms, and increasing government spending on health.

## Introduction

The health financing gap in low and middle-income countries (LMICs) persists ^1^. LMICs accounted for only 20% of the global spending on health in 2016 despite being home to over 80% of the world’s population and bearing the greatest disease burden ^1^. The low government spending in LMICs contributes to out-of-pocket payments (OOPs) becoming a major source of health financing ^2, 3^, accounting for almost half of the total health expenditure ^4, 5^. OOPs are payments made directly to healthcare providers by individuals at the point of care and this excludes prepayment mechanisms such as health insurance or taxes ^3^. OOPs, represent direct costs of care associated with disease management ^6, 7^ and they can be officially stipulated fees and sometimes unofficial or what is referred to as informal payments ^2^.

Informal payment can be defined as, “a direct contribution, which is made in addition to any contribution determined by the terms of entitlement, in cash or in-kind, by patients or others acting on their behalf, to health care providers for services that the patients are entitled to” ^8^. Some of the difficulties associated with studying informal payments include being deemed illegal in some countries thus making them a sensitive research topic ^9, 10^. This is compounded by the fact that some patients are unable to differentiate between official and unofficial fees ^9, 10^, while others refuse to respond to questions on informal payments ^9, 10^. All these factors make it challenging to estimate the magnitude and frequency of informal payments ^9^.

Despite the challenges of measuring informal payments, evidence shows that they are a common phenomenon in many countries ^9, 10^. They comprise a significant share of OOPs, accounting for 10% to 45% of total OOPs for healthcare in low-income countries ^10, 11^. Informal payments have also been reported to account for a substantial proportion of health financing resources in countries in transition ^10^. They have been argued to impede healthcare reforms ^9, 11^, and reduce the efficiency and quality of care ^9, 12^. They also limit access to care especially among the poorest and can result in catastrophic healthcare expenditure that pushes households into poverty ^10, 13^. The occurrence of informal payments has been linked to various factors. On the supply side, informal payments have been associated with inadequate funding of the health sector ^9^, limited transparency and accountability ^10, 14^, and low/irregular remuneration of staff ^10, 15^. On the demand side, patients pay informally to access care ^16, 17^, jump queues ^18^, and receive better quality services ^17, 19^. Contextual factors such as perceptions of high levels of corruption in the public sector ^14^, distrust in public institutions ^10, 11^, and norms of gift-giving also influence informal payments ^10, 14^.

Informal payments are common in almost all African countries ^13^. The 2016/18 Afrobarometer survey - a nationally representative survey that provides data on citizens' experiences and perceptions of corruption across African countries - showed that more than one in four people who sought public services such as health services and education paid a bribe. This amounted to approximately 130 million people in 35 African countries ^20^. The nature and level of informal payments can be quite specific to the health system, socio-cultural, economic, and political context. While several reviews have sought to synthesize evidence on informal payments ^10, 12, 21^, none provide a comprehensive review of informal payment practices in the SSA context.

This systematic literature review aimed to synthesize the existing evidence on the prevalence, characteristics, reasons, associated factors, and the impact of informal payments for healthcare in SSA. Findings from this review may help policymakers to gain a better understanding of informal payments and point to a range of factors they could address when developing interventions to curb informal payments. This is crucial as many SSA countries implement strategies to enhance financial risk protection as they progress towards attaining universal health coverage (UHC). This article is reported in line with the Preferred Reporting Items for Systematic Reviews and Meta-Analyses (PRISMA) guidelines ^22^.

## Methods

### Search strategy

To identify relevant literature, we searched PubMed, African Index Medicus, Directory of Open Access Journals, and Google Scholar databases. The search terms were developed with reference to the search strategies used in recent literature reviews on informal payments for healthcare ^10, 12, 21^. The main search term was “informal payment/fee/charge/expenditure” and its synonyms, that is, unofficial, illegal, illicit, envelope, under-the-table, under-the-counter, and solicited payments/fee/charge/expenditure, or bribe or corruption. These terms were combined with “health” and the list of SSA countries where applicable. The databases were last searched in August 2021. The search strategies for each database can be found as extended data ^22^.

Bibliographies of included articles were also searched to identify any relevant articles. Additionally, grey literature was searched for using free text searches on Google and websites of organizations that publish on various aspects of corruption in the health sector such as Transparency International, World Bank, World Health Organization, United Nations Development Fund, and Abdul Latif Jameel Poverty Action Lab.

### Eligibility criteria

The inclusion criteria were any empirical studies on informal payments conducted in SSA, regardless of the study design, published in any year, and in the English language. The exclusion criteria entailed reviews, editorials, and conference presentations. EK screened the articles at all levels: title, abstract and full text. Articles selected for inclusion in the review were discussed and agreed upon in consultation with the co-authors.

### Quality appraisal of included studies

The quality of qualitative studies was appraised using the critical appraisal skills program (CASP) checklist for qualitative research ^23^; while the quality of quantitative studies was assessed using the appraisal tool for cross-sectional studies (AXIS) ^24^. Mixed methods studies were appraised using both appraisal tools.

### Data extraction and analysis

Data were extracted using tables in Microsoft Excel version 16 and this entailed general study characteristics ( Table 1) and findings. Due to the variation in approaches to measuring the prevalence of informal payments across countries, a meta-analysis of quantitative data was not appropriate. We, therefore, conducted a narrative synthesis of the findings, exploring similarities and differences across the studies and contexts ^25^. A modified framework analysis approach was conducted for qualitative studies. This entailed familiarisation with the data, identification of themes, indexing data based on the themes, charting the data for comparisons, interpreting the data while exploring for relationships between concepts ^26^.

**Table 1.** General description of studies included in the review

Category	Sub-category	No.	Study reference
Publication type	Journal article Report	20 3	13, 31– 35, 36– 49 20, 50, 51
Year of publication	After 2015 2011–2015 2006–2010 2001–2005 1995–2000	10 7 5 0 1	13, 20, 32, 34, 37, 46– 50 31, 38, 40– 42, 44, 51 33, 35, 36, 39, 45 43
Data collection year	After 2015 2011–2015 2006–2010 2001–2005 1995–2000 Not clear	4 7 5 2 4 4	20, 34, 48, 49 13, 32, 34, 44, 46, 47, 51 13, 35, 37, 41, 42 31, 39 31, 33, 43, 50 36, 38, 40, 45
Country income level (2021 World Bank classification)	Low-income Lower-middle-income Upper-middle-income	10 15 3	13, 20, 31, 39, 40, 43, 44, 46, 47, 50 13, 20, 32– 38, 41, 42, 45, 48, 49, 51 13, 20, 31
Number of countries in each study	Single country Multi-country	20 3	32– 51 13, 20, 31
Sub-Saharan Africa Region	East Africa West Africa Central Africa Southern Africa	12 8 4 5	13, 20, 31, 34, 35, 39, 41– 43, 45, 49, 51 13, 20, 32, 36, 40, 44, 47, 50 13, 20, 37, 46 13, 20, 31, 33, 48
Type of study design	Quantitative Qualitative study Mixed methods	11 7 5	13, 20, 35– 37, 39– 41, 48, 49, 51 32– 34, 42, 44, 45, 50 31, 38, 43, 46, 47
Study participants	Healthcare workers Patients Households General public/ community members Policymakers	16 8 7 5 2	31– 34, 36– 38, 40– 47, 49 32, 33, 36, 37, 41, 43, 48, 50 13, 20, 31, 35, 36, 39, 51 31, 33, 40, 43, 44 38, 44

## Results

### Search results

**Figure 1.**
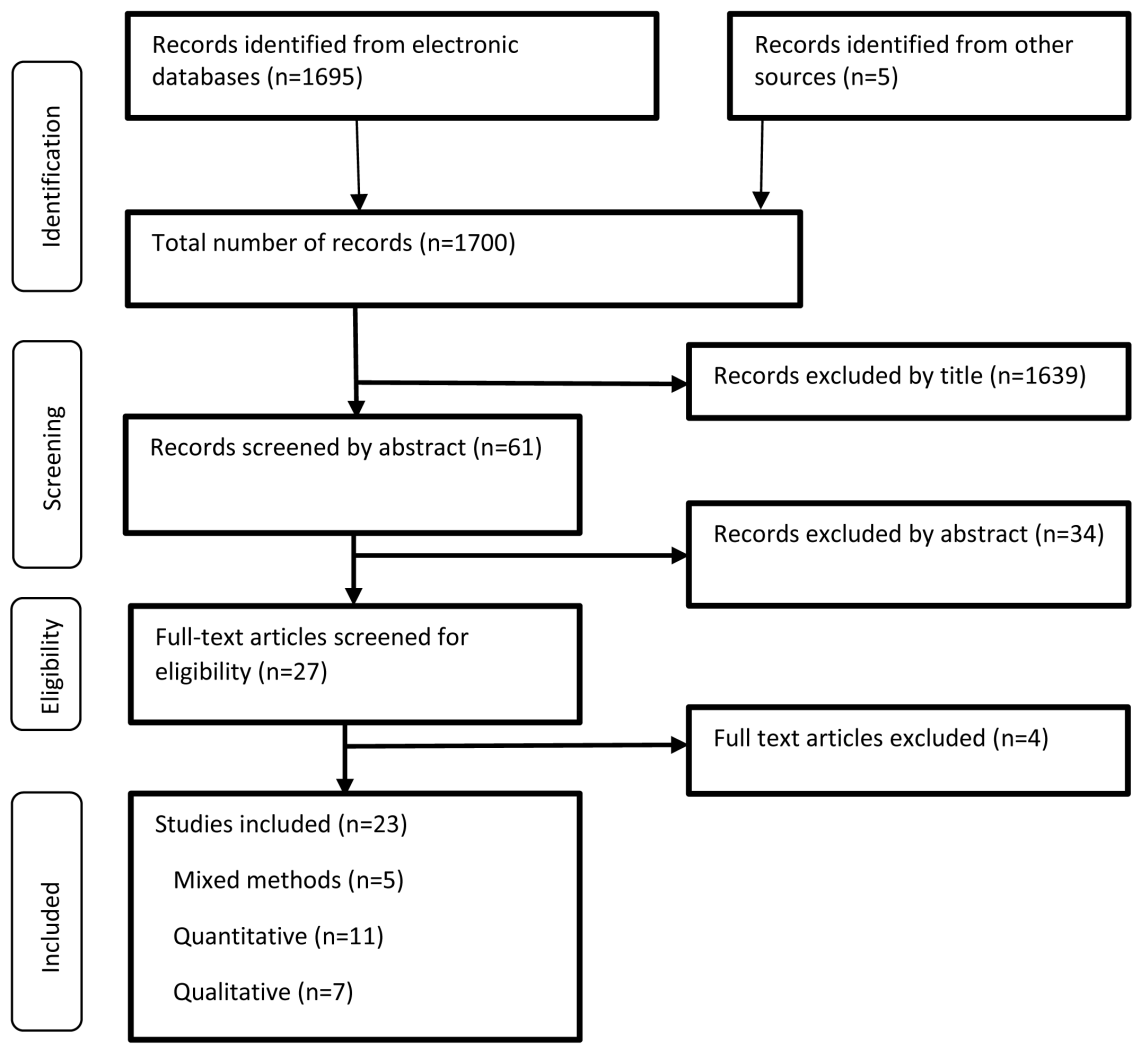
Study selection process adapted from the PRISMA 2009 flow diagram ^52^.

The literature search retrieved a total of 1700 articles which were exported into Endnote X7. Articles were screened and excluded by title, abstract, and full text respectively. Articles excluded after full text review focused on other forms of corruption other than informal payments ^27– 29^, or informal payments were combined with other payments ^30^. Overall, 23 articles were included in this review; 20 peer-reviewed articles and three grey literature. Figure 1 illustrates the study selection process.

### Study characteristics

The majority of studies (n=12) were conducted in East Africa while Central Africa had the least number of studies (n=4) ( Table 1). Three of the studies were conducted in multiple countries; one study used data from round 3 and 5 of the Afrobarometer survey conducted in 18 and 33 countries, respectively ^13^, while the second study reported findings from rounds 6 and 7 of the Afrobarometer survey conducted in 36 and 35 countries, respectively ^20^. The third multi-country study was conducted in seven countries of which two were from Africa (Uganda and South Africa) ^31^. Most studies (n=7) were conducted between 2011 and 2015. Five studies used mixed methods, eleven were quantitative, and seven were qualitative. The studies were conducted with a diverse group of participants with the majority being healthcare workers, patients, and households. Most studies assessed informal payments for health services in general while seven studies looked at informal payments for specific services, that is, maternal and child health services ^32– 35^, emergency services ^50^, malaria treatment ^36^, and HIV services ^37^.

### Prevalence of informal payments in SSA

Informal payments are a common phenomenon across East, West, Central, and Southern Africa but there was a notable variation in the prevalence across these regions ( Table 2).

**Table 2.** Prevalence of informal payments reported in cross-sectional studies

Author & country	Data collection year	Sample size and study population	Metric	Prevalence
Papers based on Afrobarometer surveys				
Pring & Vrushi ^20^; 35 African countries	2016–2018	47,000 households	The proportion that gave a gift/paid a bribe/did a favor to get services at a public health center or clinic in the past 12 months	1.0–50.0% mean:14.0%
Kankeu & Ventelou ^13^; 33 African countries	2011–2013 2005–2006	51,605 households (33 countries) 25,397 households (18 countries)	The proportion that paid a bribe, gave a gift or did a favor to government officials to get treatment at a local health clinic or hospital in the past 12 months	0.4-51.3% 2.9-47.8%
Studies based on patient/household reports				
Masiye *et al*. ^48^; Zambia	2018	1900 patients	The proportion that made any payments for healthcare services received at public primary health facilities on the survey day	6.2%
Oduor ^51^; Kenya	2012	183 households	The proportion that paid informal payments at public health facilities	10.0% (inpatient care) 8.0% (outpatient care)
Kruk *et al*. ^35^; Tanzania	2007	1322 women	The proportion that paid provider payments for free facility delivery services at government health facilities within the 5 years before the survey	84.6% (dispensary) 35.7% (health centers) 30.0% (hospitals)
Lindkvist ^41^; Tanzania	2007	3494 patients	The proportion that reported that healthcare workers at public and faith-based facilities accept informal payments	12.0%
Kankeu *et al*. ^37^; Cameroon	2006–2007	1637 HIV patients	The proportion that made informal payments for consultation with a doctor at public and private facilities on the survey day	3.1%
Paredes-Solís *et al*. ^31^; Uganda and South Africa	1998 2003	18,412 households (Uganda) 5,490 households (South Africa)	The proportion that made payments directly to healthcare workers at government health facilities	28.0% 1.0%
Hunt 2010 ^39^; Uganda	2002	12,000 households	The proportion that had paid a bribe at a public or private health facility in the past three months	17.0% (public sector) 11.0% (private sector)
Studies based on health workers reports				
Binyaruka *et al* ;Tanzania ^49^	2019	432 health workers	The proportion that had ever asked for/been given informal payment/ bribe from clients at public primary care facilities	27.1%
Maini *et al*. ^46^; Democratic Republic of Congo (DRC)	2014	406 nurses	The proportion that received informal payments/gifts from patients at public primary care facilities in the last month	16.8%
Bertone & Lagarde ^47^; Sierra Leone	2013–2014	266 health workers	The proportion that received gifts and payments from patients in the past month at public primary care facilities	74.0%
Akwataghibe *et al*. ^38^; Nigeria	not stated	69 healthcare workers	The proportion that accepted gifts and informal payments from patients at public health facilities in exchange for priority treatment	33.4%

***Prevalence from Afrobarometer studies.*** The most comprehensive data comes from a series of Afrobarometer surveys ^13, 20^. Round 7 (2016-18) conducted in 35 African countries showed that between 1% (Botswana) and 50% (Sierra Leone) of survey respondents had given a gift/paid a bribe/done a favor to get services at a public health center or clinic in the 12 months preceding the survey. Southern Africa countries accounted for more than half (8/15) of the countries with a prevalence of informal payments that was less than 10% while most of the countries with a prevalence above 20% were from Western Africa (4/10) followed by Central Africa (3/10) ^20^. Consecutive rounds of the Afrobarometer survey showed indications of increasing prevalence over time in over half (18/30) of the SSA countries that took part in both round 6 (2014-15) and round 7 ^20^. Similar trends were seen in perceptions of general corruption in the public sector, with 55% of citizens surveyed in 35 African countries in round 7 feeling that corruption was getting worse.

***Prevalence from other studies.*** Other cross-sectional studies also demonstrated considerable variation in the prevalence of informal payments across 9 settings in terms of both the proportion of patients reporting paying them and the proportion of health workers reporting receipt ( Table 2).

### Characteristics of informal payments

These entailed who initiated, the type, the timing, and the amount of informal payment paid.

***Initiation of informal payments. *** Both healthcare workers and patients initiated informal payments. Most studies where households or patients were interviewed reported that healthcare workers usually made demands for informal payments ^13, 31, 32, 43, 51^. However, in Angola, some women offered informal payments to receive pregnancy and childbirth services before demands were made hoping it would reduce the amount of money paid informally or to ensure in-kind payments would suffice ^33^. A qualitative study conducted with healthcare workers in Tanzania also reported that informal payments were initiated more often by patients than providers because patients felt they needed to pay informally to receive quality services ^45^.

***Type of informal payments.*** Informal payments made in cash ^33, 36– 39, 42, 43, 45^ were more common than those made in kind ^33, 38, 46, 47^. Informal payments were charged in addition to other fees or as standalone fees ^43^. For example, in Democratic Republic of Congo (DRC), in-kind payments came often in form of food, soap, or fabric ^46^, and in Sierra Leone, this comprised poultry, food, and charcoal ^47^.

***Timing of informal payments.*** Informal payments were made before ^32, 50^ or after service delivery ^33, 42, 50^. For example, relatives of patients seeking emergency services in Niger reported making informal payments before service provision following demands from healthcare workers and after service provision as a sign of gratitude ^50^. Some women in Angola reported that they would have preferred if the midwives delivered care first before asking for informal payments ^33^.

***The amount of informal payment.*** In total, eight studies assessed the amount of informal payments made. These studies were based on reports made by patients (n=2), households (n=2), healthcare workers (n=2), both household and patients (n=1), and the community (n=2) ^46, 47^. Regarding health worker reports, a survey conducted in DRC showed that they earned a mean income of $9 per month from informal payments ^46^ while in Sierra Leone informal payments accounted for 5% of total revenues for community health assistants and nurses ($11.8) and maternal and child health aides ($8.2) and 3% for community health officers ($9.42) per month ^47^. In terms of patient reports, for example, informal payments were the second key contributor to healthcare costs after transport costs in Tanzania accounting for 26.6% (1668 TZS (95% confidence interval [CI]: 931–2405)) of facility delivery costs at government facilities despite deliveries being exempt from user fees ^35^.

Regarding the type of service, in Kenya for example, informal payments varied depending on the family planning method. Despite being officially free, informal payments were required, with higher amounts charged for long-acting methods ^34^. Similarly, in Angola, informal payments were higher for cesarean sections compared to normal deliveries, even though cesarean sections were exempt from user fees ^33^.

### Reasons for paying informal payments

Patients or their relatives made informal payments for treatment to be initiated ^32, 50^, to receive both minor services such as bedpans ^42^, injections ^51^, or vaccinations ^33^; and major services, such as surgeries ^32, 42^. Informal payments were also made to receive drugs that were supposed to be provided for free ^32– 34, 42, 44, 48, 51^, and to obtain medical record books and reports ^48^. In Tanzania, some healthcare providers feigned stockouts of commodities and sought money from patients disguising to purchase the commodities from the private market on their behalf ^42^. Informal payments were also made to enable patients to skip queues ^33, 42, 45^ in an effort to get services more quickly ^33, 45, 51^. Some patients made informal payments hoping to receive higher quality services in return ^32, 43, 45, 51^. In extreme cases, informal payments were made to enable patients to gain access to the health facility in Niger ^50^, to obtain meals in Kenya ^51^, and for family members to see the newborn baby for the first time in Benin ^32^. Informal payments were also made to express gratitude in Angola, Tanzania, and Nigeria ^33, 34, 38, 42, 43^. Qualitative studies showed that some healthcare workers in Nigeria and Tanzania perceived informal payments as an acceptable practice and as gifts to show appreciation for their work ^38, 42^.

### Patient factors associated with informal payments

These comprised socioeconomic characteristics, health status, and social connections ( Table 3).

**Table 3.** Patient factors associated with informal payments

Patient factors	Number of citations	Study reference
**Socioeconomic characteristics**		
Age	2	37, 48
Marital status	1	37
Employment status	1	37
Income/wealth	7	13, 33, 37, 39– 41, 46
Household head	1	31
Residence (rural/urban)	3	36, 37, 48
Distance to the health facility	1	48
Awareness of service entitlements and fees	3	32, 34, 46
**Health status**		
Self-rated health	1	37
Change in health status e.g. during pregnancy/labor	1	33
**Social connections**		
Absence of connections with health facility staff	1	50

***Socioeconomic characteristics.*** People who were not aware of service entitlements and fees ^32, 34, 46^, married people ^37^, and those from male-headed households, which were probably less vulnerable than female-headed households ^31^, were more likely to make informal payments while older people ^37^ were less likely to pay informally. Regarding the amounts paid, the employed ^37^, older patients, people traveling long distances to health facilities ^48^, and those living in urban areas incurred higher amounts of informal payments ^36, 37^. However, in Zambia patients who sought services at rural compared to urban primary health facilities paid higher amounts of informal payments ^48^.

There were mixed findings on whether informal payments were more common among the rich or the poor. However, there seemed to be stronger evidence to support the latter. The prevalence was higher among the poor in almost all of the 33 countries that took part in round 5 of the Afrobarometer survey as evidenced by concentration indices ranging from -0.356 to 0.099 ^13^. Nonetheless, two nationally representative surveys conducted in Uganda and Cameroon ^37, 39^ reported that the rich were more likely to pay informal payments than the poor. Data from round 3 of the Afrobarometer survey conducted in 18 African countries also showed that healthcare workers demanded informal payments from the poor more than the rich (concentration indices ranging from -0.277 to 0.083) ^13^. However, a quantitative study that used a rating scale ranging between 0 (not at all acceptable) and 10 (completely acceptable) showed that in Togo, physician requests for informal payments were perceived to be more acceptable when patients were wealthy (Median (M)=6.35) than when they were poor (M=1.73) ^40^. Women taking part in focus group discussions (FGDs) in Angola reported that midwives did not solicit informal payments from the possibly well-off because they feared being reported ^33^.

Regarding awareness, qualitative findings from Benin showed that pregnant women who were not aware of the cesarean section user fee exemption policy were charged to access those services ^32^. In DRC nurses were less likely to charge informal payments in communities where people were aware of user fees out of fear of being reprimanded ^46^.

***Health status.*** Patient survey data from Cameroon showed that the incidence and amount of informal payments were higher among people living with HIV (PLWHA) who reported not taking antiretroviral therapy (ART) (7.31%) and having “poor” health status (7.24%) with the latter possibly aimed at receiving more attention from healthcare workers compared to PLWHA who reported taking ART (1.57%) and having “good” health status (1.57%) ^37^. Similarly, FGD participants in Angola reported that the amount of informal payments demanded increased remarkably if a pregnancy or labor changed from normal to complicated to the extent of forcing families to sell assets, borrow money, or beg to receive treatment ^33^.

***Social connections.*** Only one study reported on social connections. This qualitative study conducted in Niger showed that in the absence of connections (relatives, friends, and acquaintances) at the health facility, patients or their relatives had to pay informal payments to various cadres and non-clinical staff to access services ^50^.

### Supply-side factors associated with informal payments

These entailed healthcare workers, health facility, and system-level characteristics ( Table 4).

**Table 4.** Supply-side factors associated with informal payments

Supply-side factors	Number of citations	Study reference
**Healthcare worker characteristics**		
Age	2	46, 49
Cadre	7	32, 33, 38, 42, 45, 49, 50
Health facility manager/in-charge/head of department	2	47, 49
Consultation venue i.e. health facility/healthcare workers residence	1	43
Salary (amount and timeliness)	8	32– 34, 37, 40, 45, 49, 50
Absence of allowances e.g. transport, risk	1	45
**Health facility characteristics**		
Level of facility	5	34– 36, 47, 48
Facility ownership (public/private for profit/private non-profit)	4	34, 37, 39, 42
Facility location (rural/urban)	2	47, 48
Waiting times	3	31, 37, 48
Task shifting	1	37
Poor working conditions	1	45
Number of healthcare workers	2	45, 46
Lack of/stock out of essential drugs	2	13, 48
Presence/absence of official charging policies	3	39, 43, 50
Accountability mechanisms for user fees	1	46
Supervision/oversight over health worker behavior	2	33, 49
Poor health facility management	1	41
Engagement in informal charging/corruption by senior staff/facility managers	2	34, 45
Action against corrupt practices	1	32
**System-level characteristics**		
Corruption among top health sector management	1	45
Wide-spread corruption in the public sector	2	40, 45
Health worker post rotations	1	44

***Healthcare worker characteristics.*** Healthcare workers of all cadres charged informal payments from specialists ^42, 45, 49^, doctors ^42, 45, 49^, nurses ^33, 42, 45^, midwives ^32, 33^ to community health extension workers ^38^, medical assistants ^42, 45^ and medical students ^50^. In Sierra Leone, health facility managers/in-charges were almost three times more likely to receive gifts from patients compared to other staff (odds ratio [OR]=2.731 (1.139) P<0.05) ^47^. Similarly, in Tanzania, departmental heads were more likely to engage in informal charging (adjusted OR [AOR] 1.72 (CI: 1.15–2.57) P<0.001) ^49^. Doctors and specialists in Tanzania also had a higher likelihood of charging informal payments ^49^ and were reported to charge higher amounts compared to nurses or medical assistants ^42, 45^. In Uganda, higher amounts were paid if patients went to consult healthcare workers at their place of residence ^43^. Informal payments were less likely among health workers who were older compared to younger ones ^46, 49^.

Informal social networks within and across cadres facilitated informal charging in some health facilities in Tanzania and Benin. Healthcare workers in Tanzania for example reported that informal payments were shared mainly across cadres. In some instances, there was overt cooperation across cadres to solicit informal payments ^42^. Similarly, women who paid informally for cesarean section services in Benin reported that the midwives told them they would share the money with the other midwives, doctors, and other healthcare workers ^32^. However, in one Tanzanian study, most healthcare workers felt that informal payments were not allocated fairly ^42^. In this case and in the absence of rules on how to share informal payments, healthcare workers especially lower cadres, bargained to increase their share of the informal payment by lowering the quality of care, for example by giving less attention to patients who had bribed doctors ^42^.

Informal payments were common among healthcare workers who received low ^32– 34, 37, 40, 45^ and irregular salaries ^33, 34, 50^ and less likely with increased health worker perception that benefits and entitlements were provided on time ^49^. Healthcare workers reported that their salaries were inadequate to meet their basic needs ^34, 45^ and for the level of effort and skill required of them ^34^. Laypeople and health professionals in Togo found it more acceptable (M=4.89) for physicians to request informal payments when they were underpaid than when they were well paid (M=3.06) ^40^. The latter is supported by FGD findings from Tanzania where healthcare workers reported that informal payments were a coping strategy for their low salaries and lack of allowances ^45^. Some women in Angola also acknowledged that the prolonged civil war which worsened everyone’s socioeconomic situation contributed to the charging of informal payments by midwives. However, some of the women also felt that their continued compliance with demands for informal payments perpetuated the practice ^33^.

Despite complaints of low salaries, some healthcare workers in Tanzania perceived charging of informal payments as a form of corruption ^42^ which would damage their reputation and that of the health facility ^45^. Some healthcare workers were also discouraged from charging informal payments because patients felt empowered to manipulate them after paying a bribe and this made healthcare workers feel humiliated and enslaved to patients ^52^. This was in addition to some patients expecting to receive better treatment during subsequent visits ^42^. In Kenya, healthcare providers acknowledged that charging informal payments was bad practice but some did not perceive informal payments as a challenge as long as the healthcare provider was willing to forgo the payment and offer health services if they discerned the patient did not have the ability to pay ^34^. Healthcare providers were conflicted between meeting their basic needs for survival while also taking into account the financial hardship of the patients ^34^.

***Health facility characteristics.*** In terms of facility management, informal payments were more likely to be made at facilities that lacked official charging policies ^39, 50^ and oversight over healthcare workers behaviors’ ^33^, and where senior staff and facility managers were reported to be corrupt or to engage in charging of informal payments ^34, 45^. Informal charging was also more likely to take place at facilities with poor working conditions, staff ^45^ and medicine shortages ^13^, long waiting times ^31, 37, 48^, facilities that did not implement task shifting practices ^37^, and urban facilities ^47, 48^. With regards to waiting times and task shifting practices (delegation of subsequent consultations from doctors to nurses), patient survey data from Cameroon showed that patients seeking HIV care at facilities with long waiting times had a higher risk of paying informally (AOR 95% CI 3.68 (1.27–10.68)) P ≤ 0.05 while task-shifting of HIV services reduced the risk of incurring informal payments (AOR 95% CI 0.31 (0.11–0.90)) P ≤ 0.05 ^37^.

Informal payments were less likely to be made at facilities where patients paid official fees ^39, 43^, facilities with accountability mechanisms for the user fees ^46^, supervision throughout ^49^ and where action was taken against corrupt practices ^32^. The likelihood of paying informally was also less at facilities with more staff ^46^ and those reported to be well-managed ^41^.

In terms of facility ownership, there were mixed findings on whether informal payments were more prevalent in the public or private sector. In Uganda, the prevalence (17%) and amount of bribes ($6.06) paid by individuals in the public health sector were higher than the prevalence (11%) and the amount paid ($5.26) in the private sector (non-mission facilities) ^39^. Similarly, healthcare providers in Kenya reported that informal payments were more likely to occur in government facilities partly due to lower wages in the public sector and lower risk of facing consequences if found charging informal payments ^34^. On the contrary, a survey done with PLWHA in Cameroon showed that the incidence and amount of informal payments charged in private for-profit facilities were higher than in both public hospitals and non-profit hospitals ^37^.

There were mixed findings regarding informal payments across different levels of healthcare. For example, a patient survey done in Zambia found that informal payments were more common at public hospitals (9.7%) compared to public health centers (5.8%) ^48^. On the other hand, in Tanzania, informal payments were higher at government dispensaries (84.6%) compared to government health centers (35.7%) and hospitals (30.0%) ^35^. In terms of amount, surveys done in Nigeria ^36^ and Zambia ^48^ showed that informal payments for malaria treatment and primary health services respectively were higher in public hospitals compared to healthcare centers. However, in Sierra Leone healthcare providers working in higher-level primary health care (PHC) facilities (community health centers and community health posts) received less income from gifts compared to those working in lower-level PHC facilities (maternal and child health posts) ^47^.

***System-level characteristics.*** Corruption in the public sector and staff transfers were reported to encourage the charging of informal payments. Some of the healthcare workers taking part in FGDs in Tanzania reported that corruption among officials at the top management level in the health sector and widespread corruption in the entire public sector promoted the charging of informal payments ^45^. These findings are supported by a study done in Togo where laypeople and health workers found it more acceptable (M=4.47) for physicians to ask for informal payments when it was a common practice in other local public institutions than when the practice was rare (M=3.61) ^40^. 

In terms of human resource management practices, FGDs in Sierra Leone showed that routine rotations of healthcare workers across facilities led to an increase in charges with the new healthcare workers reintroducing charges for free health care ^44^.

### Impact of informal payments on the quality of care

Informal payments were associated with negative patient experiences with health services ^31, 39^. For example, household survey data from Uganda showed that patients who paid informally were less likely to report that they were satisfied with the health services they received (AOR 0.27, 95% CI 0.24-0.29) ^31^. Paying informally was associated with longer health facility visits with patients and members of the public who used government services and paid bribes reporting having spent more time to get the services needed (AOR, 2.04, 95% CI 1.89-2.22) ^31^. In Tanzania, direct observation of healthcare workers during consultation showed that those who had a higher probability of accepting informal payments put in less effort for patients who were classified as weak in comparison to other healthcare workers. This indicated that they did not vary their effort based on the patient’s medical condition and therefore did not provide care based on patients’ needs ^41^.

In terms of safety, in Tanzania, FGDs with healthcare workers revealed that some of their colleagues deliberately prolonged waiting times for surgeries. This was aimed at making patients desire to pay for quicker services at the public facility or the doctor’s private practice ^42^. Such delays could potentially put the patient’s life at risk. Furthermore, some healthcare workers claimed that some of their colleagues provided very low-quality care, first, to hint to the patients that the quality of care would be very low if they did not give informal payments; and secondly when they felt that there was an unfair allocation of informal payments ^42^.

In some Tanzanian health facilities, the provision of high-quality services was perceived to have resulted from having received informal payments. This could have forced non-corrupt healthcare workers to lower the quality of care to protect themselves from being labeled as corrupt ^42^.

### Impact of informal payments on equity

Demands and actual payment of informal fees disproportionately affect the poor according to rounds 3 and 5 of the Afrobarometer survey ^13^. Informal payments perpetuated health inequities in access to care. Qualitative findings from Uganda, Angola, and Kenya showed that some people were forced to delay ^34^ or forgo care because they could not afford to pay informal payments ^33, 43^, leading to unintended consequences such as unwanted pregnancies ^34^. Informal payments also prevented access to specialized services at public hospitals in urban areas in Tanzania ^45^. The high prevalence of informal charges at dispensaries in a rural district in Tanzania was also thought to contribute to low facility delivery rates (40%) ^35^.

Respectful service delivery was dependent on an individual’s ability to pay informally ^33, 43^. For example, community members in Uganda reported that the inability to pay informal payments led to healthcare workers being reluctant and impolite ^43^ while in Angola it led to negligence or denial of care and in extreme cases obtaining “labor on credit” by pledging to pay later ^33^. In Uganda, the ability to pay informally led to obtaining cooperation from healthcare workers ^43^ and getting “royal treatment” in Angola ^33^.

In some instances, informal payments led to the development of negative attitudes towards healthcare workers. For example, FGD participants in South Africa and Uganda reported feeling angry ^31^ while women in Angola reported feeling anxious when healthcare workers demanded informal payments ^33^. Healthcare workers in Kenya reported that informal payments could demoralize patients, especially where they incur costs for services they are aware should be provided for free ^34^. Being cognizant that informal payments were an access barrier to the poor, some healthcare workers in Tanzania and DRC reported feeling uncomfortable charging informal fees ^45, 46^.

## Discussion

Several studies ranging from large-scale nationally representative surveys to in-depth qualitative studies have shown that informal payments for healthcare are a common phenomenon in SSA regardless of the health service, facility level, and sector. Informal payments have also been reported to be prevalent in other regions such as Central and Eastern Europe, Asia, and South America ^10, 12, 21^.

Informal payments limited access with the inability to pay associated with disrespectful care ^33, 43^, delayed care-seeking ^34^, and foregone care ^33, 43^. Informal payments also incentivized some healthcare providers to lower their quality of care to induce patients to pay informally to receive better services ^42^. The negative impact is of particular concern especially for the poor because they bear the greatest burden of informal payments ^13^. Evidence from both low and high-income countries shows that informal payments are inequitable and regressive ^53^. They have been reported to lead to delayed hospitalization, use of savings, borrowing, and sale of assets to acquire resources to pay informally in countries such as Tajikistan, Hungary, Poland, and Romania ^53– 55^.

Mostly, healthcare workers rather than the patients initiated informal payments ^13, 31, 32, 43, 51^. Patients paid informally, before care, mainly to access drugs and services, many of which should have been provided for free ^32– 34, 42, 48, 51^. Some patients also made informal payments after service delivery as gifts to express gratitude ^42, 50^. Most informal payments were made in cash ^33, 36– 39, 42, 43, 45^ rather than kind ^33, 38, 46, 47^. It has been argued that it is difficult to differentiate voluntary gifts from solicited payments in the health sector. This is compounded by the fact that some patients may offer gifts out of fear of not receiving good healthcare services ^8^.

From the demand side, low socio-economic status ^13^ and lack of awareness of user fees ^32, 34, 46^ were some of the key characteristics associated with a higher likelihood of paying informal payments. Other than a socio-economic disadvantage, inequities in informal payments in most African countries have been attributed to disparities in supply-side factors, such as lack of drugs, long waiting times, shortage of doctors, and regional differences within countries that disadvantage the poor forcing them to make informal payments to obtain better quality care ^13^.

Some of the notable supply-side factors that increased the likelihood of paying informally were low healthcare worker salaries ^32– 34, 37, 40, 45, 49, 50^, absence of official fees ^39, 50^, and perceptions of widespread corruption in the public sector ^40, 45^. Low and irregular healthcare worker salaries could be associated with low government spending on health. For example, per capita, government health expenditure was very low in countries with the highest prevalence of informal payments, Sierra Leone ($23), Liberia ($11), and DRC ($7) compared to countries with a low prevalence of informal payments, Botswana ($564) and Eswatini ($427) ^56^. Low healthcare worker salaries have also been linked with the charging of informal payments in transition countries such as the Russian Federation, Ukraine, and Georgia due to economic difficulties that led to reduced government spending on health ^8, 57– 59^. Informal payments were less likely at health facilities where patients paid official fees ^39, 43^. Similar findings have been reported in transition countries such as Kyrgyzstan, Cambodia, and the Kyrgyz Republic where informal payments reduced following the introduction of co-payments alongside other initiatives ^14, 60, 61^. However, user fees reduce the utilization of health services especially among the poor ^62, 63^, and therefore formalization of user fees in SSA would also require the implementation of effective exemption policies for the poor and other vulnerable groups ^64^. The effectiveness of formalization of user fees in reducing informal payments also warrants further investigation since the effects were not sustained in some transition countries such as Kyrgyzstan ^61^.

This review identifies some distinctive features of informal payments in SSA. First, regional differences observed in the occurrence of informal payments can partly be associated with variation in the level of perceived corruption in the public sector. In countries with the highest prevalence of informal payments, a higher proportion of households reported paying a bribe to use public services compared to countries with the lowest prevalence ^20^. Secondly, the presence of political instability appeared to contribute to the variation in the prevalence of informal payments in SSA. Countries with the highest prevalence of informal payments, Sierra Leone, Liberia, and DRC had also faced political instability in recent years. Constant conflict and insecurity in DRC have been linked with underfunding of public services and this might have encouraged informal charging ^20^. Third, two qualitative studies revealed the existence of informal social networks that promote informal charging among and across cadres. Informal social networks have been linked to the development of strong moral obligations such as expectations to assist others within the network and to return favors that may surpass any existing formal rules ^65^. If left unchecked informal social networks among healthcare workers may continue to promote the charging of informal payments.

### Limitations

This review had some limitations. One, the literature search was limited to studies published in English. Secondly, due to the vast nature of grey literature, some insights on informal payments in SSA might have been missed. Thirdly, these findings can only be applied to similar low and middle-income countries with caution since factors affecting informal payments vary across contexts.

Some gaps were identified in the literature. There was limited information on the amount of informal payments incurred, variations in informal payments across various levels of care, and strategies used to tackle informal payments and their effectiveness. These are all potential areas for future research. There is also a need for further investigation on informal payments across all SSA regions because of the changes in health financing as countries strive to achieve UHC.

### Policy considerations

Curbing informal payments calls for a multi-faceted approach with various short and long-term strategies because individual strategies alone cannot address the complexity of associated factors. Drivers of informal payments highlighted in this review provide some suggestions that policymakers in SSA could take into consideration and monitor to assess their effectiveness. In the short term, there is a need to enhance public awareness about official user fees, and services and population groups that are exempt from user fees. Accountability mechanisms at health facilities should also be strengthened. This could entail the establishment of safe and effective whistle-blower mechanisms for patients to report informal payment incidences and enhanced supportive supervision of health facilities. SSA governments should also increase their political commitment to fighting corruption in the health sector. In the medium to long term, there is a need for better remuneration for healthcare workers. This should be implemented alongside alternative incentive programs such as the provision of bonuses, better working conditions, and opportunities for career advancement. Increased government spending on health is also crucial as this would address healthcare worker shortages, poor working conditions, and drug stock-outs which were reported among the factors that encouraged informal payments. Equitable geographical distribution of health resources should also be ensured.

## Conclusions

Informal payments are a common phenomenon in SSA, and the highest prevalence was reported in conflict and post-conflict countries and countries where corruption was perceived to be widespread in the public sector. Various patient and supply-side factors were associated with informal payments. Patients paid informally mainly to access services and drugs which were supposed to be provided for free. There was little evidence to suggest that paying informal payments led to the provision of higher quality care. Informal payments limited access and utilization of care especially among the poor and the inability to pay led to the provision of lower-quality care.

Some of the potential strategies that policymakers can consider when developing interventions to address informal payments include enhancing patient awareness about service fees, revisiting health worker incentive schemes, strengthening accountability mechanisms, and increasing government spending on health.

## Data availability

### Underlying data

All data underlying the results are available as part of the article and no additional source data are required.

### Extended data

Harvard Dataverse: The hidden financial burden of healthcare: a systematic literature review of informal payments in Sub-Saharan Africa. https://doi.org/10.7910/DVN/NMQCSF ^22^.

This project contains the following extended data:

- Characteristics of studies included in the review_table.docx
- DataReadme_Kabia_et_al_review.txt
- Search_strategy.docx

### Reporting guidelines

Harvard Dataverse: PRISMA checklist for ‘The hidden financial burden of healthcare: a systematic literature review of informal payments in Sub-Saharan Africa’. https://doi.org/10.7910/DVN/NMQCSF ^22^.

Data are available under the terms of the Creative Commons Attribution 4.0 International license (CC-BY 4.0).
