# Supplementary material for: The hidden financial burden of healthcare: a systematic literature review of informal payments in Sub-Saharan Africa
Source: Wellcome Open Res. 2021 Nov 8;6:297. [Version 1] doi: 10.12688/wellcomeopenres.17228.1 (PMC9513412; doi:10.12688/wellcomeopenres.17228.1)
Supplement: Peer review history

# Peer review: Reviewer response for version 1

**DOI:** 10.21956/wellcomeopenres.19039.r52203

**Published:** 2022-09-26

**Authors:** Laetitia Rispel

**Affiliations:** 1 Centre for Health Policy & South African Research Chairs Initiative (SARChI), School of Public Health, Faculty of Health Sciences, University of the Witwatersrand, Johannesburg, South Africa

Thank you for the opportunity to review the manuscript entitled: ***The Hidden financial burden of health care: a systematic literature review of informal payments in Sub-Saharan Africa***

 Below, the main comments on the paper. 

**Abstract**

 I found that the abstract provides an adequate synopsis of the paper. I suggest that the first sentence in the background should remove “equitable” as informal payments limit access in general, and equity and quality in particular.

**Introduction**

 The introduction primarily focusses on informal payments from the perspective of health financing, but it is important to highlight the governance and moral dimension of so-called informal payments in Sub-Saharan Africa.

 Given that the literature review only focuses on the English literature, the authors should be more circumspect in the fourth paragraph of the introduction. Perhaps phrase the first sentence to say that: “ *Reports of informal payments are common in English-speaking African countries*”.

**Methodology**

 It would be helpful if the authors should state the period of the review early on, so that the reader is clear on this aspect.

 Although the authors noted that there was quality appraisal of the studies, it is not clear whether all the studies were of equal quality. If not, this might influence some of the conclusions reached, given the nature of the topic.

**Discussion**

 The discussion is silent on ethical values, codes of conduct of health professionals, pre-service training, and the role of the regulators (namely the health professions councils) in addressing the problem of informal payments. These aspects constitute a major omission and should be included in the discussion and as recommendations.

 The section on limitations should also highlight the importance of context, and potential social desirability bias, especially if patients were aggrieved with the quality of health services in general, and the behaviours of health workers in particular.

Are the rationale for, and objectives of, the Systematic Review clearly stated?

Yes

Is the statistical analysis and its interpretation appropriate?

Not applicable

Are sufficient details of the methods and analysis provided to allow replication by others?

Partly

Are the conclusions drawn adequately supported by the results presented in the review?

Partly

Reviewer Expertise:

Human resources for health; health systems; health policy; public health

I confirm that I have read this submission and believe that I have an appropriate level of expertise to confirm that it is of an acceptable scientific standard, however I have significant reservations, as outlined above.

# Peer review: Reviewer response for version 1

**DOI:** 10.21956/wellcomeopenres.19039.r52199

**Published:** 2022-09-08

**Authors:** Pieternella Pieterse

**Affiliations:** 1 School of Nursing, Psychotherapy and Community Health, Glasnevin Campus, Dublin City University, Dublin, Ireland

This is a timely synthesis of a particular issue that undermines healthcare delivery in SSA; informal payments. The authors provide great detail in relation to the included articles, which allows the reader to gain a fresh perspective on the publications related to this issue, regarding what has been published so far and what, where, and who have the articles focused on. I felt that the rationale and objectives for this research were clearly stated. The inclusion and exclusion criteria are well defined and the search strategy seems suitable. The Prisma diagram is clear. The presentation of the findings is good, there are a few places where the sentence flow is a little off, but these instances are minor (see list below). 

 The conclusions are well drawn and supported by examples from the literature, and I welcome some additional examples of informal payment cases from other parts of the world. The short section on solutions to this problem is not strictly necessary from a methodological perspective but nevertheless welcome, as readers tend to be left with a sense of despair reading the evidence contained in this review. 

 Well done to the authors!

 I support this article's indexing with very minor revisions - see my suggestions (in italic) below: 

**On page 11**: In Tanzania, direct observation of healthcare workers during consultation showed that those who had a higher probability of accepting informal payments put in less effort for patients who were classified as weak in comparison to other healthcare workers. This indicated that they did not vary their effort based on the patient’s medical condition and therefore did not provide care based on patients’ needs 

*These two lines are a bit difficult to understand.*

*Perhaps provide a little more detail to flesh out the first line? Two things that are unclear are: how are 'those who had a higher probability of accepting informal payments' found to be so [in other words, how do the authors know which people (health workers?) have a higher probability of accepting informal payments?] And how are certain patients classified as 'weak'?*

*Even if you changed it to:  In Tanzania, direct observation of healthcare workers during consultation showed that, in comparison to others, those healthcare workers who had a higher probability of accepting informal payments, put in less effort for patients who were classified as weak. {but pls also explain the 'classified as weak' bit}*

**On page 11**: Respectful service delivery was dependent on an individual’s ability to pay informally. For example, community members in Uganda reported that the inability to pay informal payments led to healthcare workers being reluctant and impolite while in Angola it led to negligence or denial of care and in extreme cases obtaining “labor on credit” by pledging to pay later. In Uganda, the ability to pay informally led to obtaining cooperation from healthcare workers and getting “royal treatment” in Angola.

*I suggest rewording this paragraph slightly; perhaps combining the findings per country, as there are clearly findings from two paper, one about Uganda, the other about Angola.*

*The text here suggests that in both countries care is 'respectful' if informal payments can be made, with the actual service being described later on as 'obtaining cooperation from health workers' in Uganda, and getting the "royal treatment" in Angola; unless all these terms were used in the original paper, I would caution against describing any service that was contingent on informal payments as 'respectful service delivery' -perhaps use 'patients were treated with more courtesy' when they paid up. Perhaps also note that the Angola paper suggests the biggest difference; from negligence and denial of care to royal treatment.*^^

**On page 11: **Several studies ranging from large-scale nationally representative surveys to in-depth qualitative studies have shown that informal payments for healthcare are a common phenomenon in SSA regardless of the health service, facility level, and sector.

*The end of this sentence 'regardless... sector.' is a bit confusing - both 'health service' and 'sector' can mean so much; Do you mean regardless of the type of healthcare (primary care, maternity care, hospital, specialist etc)? And sector? Or do you mean 'regardless of whether it concerns the public or private sector'?*

**On page 11:** Other than a socio-economic disadvantage, inequities in informal payments in most African countries have been

 attributed to disparities in supply-side factors, such as lack of drugs, long waiting times, shortage of doctors, and regional differences within countries that disadvantage the poor forcing them to make informal payments to obtain better quality care

*I had to read this line four times to finally get what was meant here. I think you mean: Supply-side factors, such as lack of drugs, long waiting times, shortage of doctors... tend to disproportionately disadvantage the poor, forcing them to make informal payments to obtain better quality care.  Please consider rephrasing this line.*

**On page 12:** However, user fees reduce the utilization of health services especially among the poor, and therefore formalization of user fees in SSA would also require the implementation of effective exemption policies for the poor and other vulnerable groups

*Make sure to highlight that you are assuming that the objective is equitable health access, so I suggest adding a couple of words, such as: and therefore, reduced informal fees and continued or improved accessibility would only be achieved if formalization of user fees in SSA was combined with effective exemption policies for the poor and other vulnerable groups.*

**On page 12:** In countries with the highest prevalence of informal payments, a higher proportion of households reported paying a bribe to use public services compared to countries with the lowest prevalence.

*This sentence needs to be corrected, I presume you want to make the connection between highest corruption PERCEPTION and highest ACTUAL payment of informal fees?*

**On page 12:** Countries with the highest prevalence of informal payments, Sierra Leone, Liberia, and DRC had also faced political instability in recent years. Constant conflict and insecurity in DRC have been linked with underfunding of public services and this might have encouraged informal charging.

*I agree with the observation concerning DRC, but both Sierra Leone and Liberia have NOT been politically unstable since emerging from civil war 20-odd years ago. This makes these two outliers regarding their terrible health outcomes in countries relatively unaffected by recent conflict (but highly affected by corruption).*

**On page 12: **Third, two qualitative studies revealed the existence of informal social networks that promote informal charging among and across cadres. Informal social networks have been linked to the development of strong moral obligations such as expectations to assist others within the network and to return favors that may surpass any existing formal rules65. If left unchecked informal social networks among healthcare workers may continue to promote the charging of informal payments.

*This is really interesting and deserves to be highlighted a bit more! I know this was an observation based on only two papers, but I wonder if this can be added to the results? It deserves a paragraph that explains which papers, which contexts, etc. Or if not in the results, consider referring to it again at the end as an issue that 'warrants further exploration'?*

Are the rationale for, and objectives of, the Systematic Review clearly stated?

Yes

Is the statistical analysis and its interpretation appropriate?

Not applicable

Are sufficient details of the methods and analysis provided to allow replication by others?

Yes

Are the conclusions drawn adequately supported by the results presented in the review?

Yes

Reviewer Expertise:

Health systems, governance in the health sector, access to basic public services

I confirm that I have read this submission and believe that I have an appropriate level of expertise to confirm that it is of an acceptable scientific standard.

# Peer review: Reviewer response for version 1

**DOI:** 10.21956/wellcomeopenres.19039.r46955

**Published:** 2022-02-08

**Authors:** Péter Gaál

**Affiliations:** 1 Semmelweis University, Budapest, Hungary; 2 Sapientia Hungarian University of Transylvania, Targu Mures, Romania

This is a very well written and scientifically sound literature review, which represents an important contribution to the study of informal payment for health care. I have only minor suggestions to improve the manuscript, whose details I have provided below. My only major point is that the discussion of the policy implications of the work is limited, but I understand that the discussion of how to best address informal payment would require a paper of its own, which is not possible to fit in the scope of this paper. Once this paper is indexed, I will consider writing a commentary on this topic, if the journal is open to such an idea. :)

**Detailed comments:**

1. ad methods: Given that most of the identified records were excluded on the basis of the title alone (n=1639), some explanation on how this was done might be in order here.
2. Page 3. "On the demand side, patients pay informally to access care, jump queues, and receive better quality services.": This statement ignores gratitude payment and the role of cultural factors. I would refine the sentence by stating that the motivation for informal payments is multifaceted, and at least four main categories can be distinguished: lack of information on official entitlements (ignorance), to thank for being cured, to get adequate care, to get distinguished care, which can and do coexist in health system, albeit the share of each is debated. ^1^^,^^2^
3. Page 7. "given gift/paid a bribe/done a favor": I suggest putting a space before or after the forward slashes to avoid any confusion regarding how to read the text.
4. Page 10. „On the contrary, a survey done with PLWHA in Cameroon showed that the incidence and amount of informal payments charged in private for-profit facilities were higher than in both public hospitals and non-profit hospitals.": this is a very interesting finding regarding private sector informal payments but needs some clarification. It does matter whether these private for-profit facilities provide publicly funded services, which should be free of charge (or could be utilised with some co-payment), or the financing of these services are also private, and patients pay in addition to the official price (fee) of the service, set by the private providers themselves.
5. Page 11: The presentation of the findings regarding the impact of informal payment on the quality of care and equity is somewhat confusing. First, there are some findings regarding responsiveness, which rather fit under the quality of care subheading than under equity, if not discussed under a separate subheading. Second, the findings that certain population groups, such as the poor, receive better or worse quality of care if they pay or do not pay informally, as opposed to other population groups, such as the rich, is a question of equity, rather than quality of care per se. Further, the findings which are presented under the equity subheading are mainly related to access to care, which is a narrower concept than equity. In order to avoid these confusions, I suggest putting all of these findings under one subheading: The impact of informal payments on the quality of care, responsiveness, access to care and equity.
6. Page 12. “Similar findings have been reported in transition countries such as Kyrgyzstan, Cambodia, and the Kyrgyz Republic where informal payments reduced following the introduction of copayments alongside other initiatives.”: To my knowledge, Kyrgyzstan and the Kyrgyz Republic are the same country. :)
7. ad discussion and limitations: “Thirdly, these findings can only be applied to similar low and middle-income countries with caution since factors affecting informal payments vary across contexts” I think this assertion is too cautious and unassuming. The empirical evidence on informal payments for instance from former communist countries, such as Hungary, Slovakia, Romania, Poland, Bulgaria, Ukraine and the Central Asian Republics of the former Soviet Union, but other countries as well, such as Greece (see for instance 3,4), ^3^^,^^4^ show remarkable similarities with the African countries reviewed in this paper. In the discussion section, I would highlight these similarities, for instance regarding the motivation for informal payments, and the contextual factors, such as the low level of public financing and underpaid health care staff. This is important to realize that even seemingly very different countries can and should learn from each other. On the other hand, the financial and economic situation of these groups of countries does differ, which could be a reason why certain practices, such as informal charging, are more prevalent in African countries than in Central and Eastern European countries.
8. To the limitations section, I would add that the various findings of the study are not unequivocally robust, and there is a difference between a finding which is supported by multiple studies and a finding which is supported by only a few studies or at the extreme only one study.
9. Page 12. “There was little evidence to suggest that paying informal payments led to the provision of higher quality care. Informal payments limited access and utilization of care especially among the poor and the inability to pay led to the provision of lower-quality care.” These two assertions seem to be in contradiction with each other. If a patient is unable to pay and as a result receives lower quality of care, then those who pay have to receive higher quality, even if this higher quality is not higher than the standard level.

Are the rationale for, and objectives of, the Systematic Review clearly stated?

Yes

Is the statistical analysis and its interpretation appropriate?

Not applicable

Are sufficient details of the methods and analysis provided to allow replication by others?

Partly

Are the conclusions drawn adequately supported by the results presented in the review?

Yes

Reviewer Expertise:

Health systems, health policy, health financing, informal payments for heath care in particular

I confirm that I have read this submission and believe that I have an appropriate level of expertise to confirm that it is of an acceptable scientific standard, however I have significant reservations, as outlined above.

## References

[1] : Gift, fee or bribe? Informal payments in Hungary. *Pluto Press, Transparency International: Malta* .2006;71-73 Reference source

[2] : Fee-for-service or donation? Hungarian perspectives on informal payment for health care. *Soc Sci Med* .2005;60(7) : 10.1016/j.socscimed.2004.08.009 1445-57 10.1016/j.socscimed.2004.08.009 15652678

[3] : Patterns of informal patient payments in Bulgaria, Hungary and Ukraine: a comparison across countries, years and type of services. *Health Policy Plan* .2017;32(4) : 10.1093/heapol/czw147 453-466 10.1093/heapol/czw147 27993960

[4] : Addressing informal payments in the Greek health system. *World Health Organization, Regional Office for Europe: Copenhagen* .2018;1-32 Reference source
